# Synthesis and luminescent properties of uniform monodisperse LuPO_4_:Eu^3+^/Tb^3+^ hollow microspheres

**DOI:** 10.1098/rsos.171451

**Published:** 2017-12-20

**Authors:** Yu Gao, He Yu, Cheng Shi, Guiyan Zhao, Yanfeng Bi, Baotong Xu, Fu Ding, Yaguang Sun, Zhenhe Xu

**Affiliations:** 1 The Key Laboratory of Inorganic Molecule-Based Chemistry of Liaoning Province, College of Applied Chemistry, Shenyang University of Chemical Technology, Shenyang 110142, People's Republic of China; 2 College of Chemistry, Chemical Engineering and Environmental Engineering, Liaoning Shihua University, Fushun 113001, People's Republic of China

**Keywords:** hydrothermal, luminescence, rare earth compounds, hollow microspheres

## Abstract

Uniform monodisperse LuPO_4_:Eu^3+^/Tb^3+^ hollow microspheres with diameters of about 2.4 µm have been successfully synthesized by the combination of a facile homogeneous precipitation approach, an ion-exchange process and a calcination process. The possible formation mechanism for the hollow microspheres was presented. Furthermore, the luminescence properties revealed that the LuPO_4_:Eu^3+^ and LuPO_4_:Tb^3+^ phosphors show strong orange-red and green emissions under ultraviolet excitation, respectively, which endows this material with potential application in many fields, such as light display systems and optoelectronic devices. Since the synthetic process can be carried out at mild conditions, it should be straightforward to scale up the entire process for large-scale production of the LuPO_4_ hollow microspheres. Furthermore, this general and simple method may be of much significance in the synthesis of many other inorganic materials.

## 1. Introduction

Nowadays, rare earth luminescent micro/nanomaterials which have lots of excellent physical and chemical properties arising from their intra-4f transitions have attracted great attention from scientists and also been widely applied to lasers, displays, sensors, solar cells, electroluminescent devices and biomedical research [1–7]. Furthermore, to the best of our knowledge, the properties of rare earth luminescent micro/nanomaterials have a strong dependence on their chemical composition, size, morphology and crystallinity [8–13]. Therefore, regulating and controlling the size, shape and morphology of rare earth luminescent micro/nanomaterials are the focuses of chemistry and materials science [14,15]. Significant interest has recently been directed towards the formation of hollow spheres, which have porous shell, low density, high surface-to-volume ratio, low coefficients of thermal expansion and low refractive index, and have widespread applications in drug-delivery carriers, efficient catalysis, waste removal, sensors, active-material encapsulation, photonic crystals, batteries and so on [14–19]. Among the methods employed for the preparation of hollow spheres, template method has been widely used. Generally, the desired materials firstly are coated onto the core templates (e.g. SiO_2_ [20], carbon spheres [21], polymers [22], metal particles [23]). Then, the core templates are removed by chemical etching or thermal treatment. Among the various core templates, considerable research efforts have been devoted to using colloid polystyrene (PS) as template to synthesize hollow spheres, because they can be conveniently removed by selective dissolution in an appropriate solvent or by calcination at elevated temperature in air. To date, many inorganic hollow nano/microspheres, such as TiO_2_ [18], BaTiO_3_ and SrTiO_3_ [24], have been successfully prepared via the template-directed synthesis route using PS as template. Hence, a facile, economic and green method to synthesize rare earth hollow micro/nanomaterials for large-scale industrial preparation with defined shape and multiple properties should be highly promising.

Among all of rare earth luminescent materials, lanthanide orthophosphates (LnPO_4_) have excellent characteristics due to their low solubility in water, high chemical/thermal stability and high refractive index [2,8,14], which make them promising candidates for a variety of applications in down/up-conversion luminescence, magnets, lasers and bio-labelling [25,26]. Recently, many researches have reported on the synthesis of YPO_4_ [27], LaPO_4_ [28], CePO_4_ [29] and GdPO_4_ [14] micro/nanomaterials. Compared with a great deal of work on other orthophosphate materials, the study on the synthesis of LuPO_4_ material has rarely been reported [30–34]. LuPO_4_ is an excellent candidate for lanthanide ion substitution because of its favourable physical properties, such as high chemical stability, high melting point, high quantum yield and low toxicity. Up to now, some typical morphologies of LuPO_4_, such as zero-dimensional nanoparticles [31], microspheres [32], one-dimensional nanorods [30] and three-dimensional microtetrahedron [30], have been successfully synthesized.

In our prior study, we reported the synthesis of the monodisperse LuPO_4_ hollow spheres by using the Lu(OH)CO_3_ precursor spheres as templates through the hydrothermal process [34]. However, to the best of our knowledge, there have been few reports on the synthesis of uniform, well-dispersed micrometre-scaled rare earth-doped LuPO_4_ hollow spheres and their corresponding luminescence properties. Therein, the novel LuPO_4_ hollow microspheres with diameters of about 2.4 µm were prepared by the combination of a facile homogeneous precipitation approach, an ion-exchange process and a calcination process. The structure, morphology, formation process and luminescence properties of the as-obtained hollow microspheres were investigated in detail. Moreover, the special structural geometry and excellent photoluminescent properties of the as-obtained LuPO_4_ hollow microspheres will have promising potential to serve as solid-state lasers and display devices. Furthermore, this synthetic methodology may be promising for the synthesis of other hollow spherical materials because of its simplicity and the low cost of the starting reagents.

## 2. Experimental section

### 2.1. Materials

The rare earth oxides Ln_2_O_3_ (99.99%) (Ln = Lu and Eu) and Tb_4_O_7_ (99.99%) were purchased from GZSUNKO new material Co., Ltd. Other chemicals were purchased from Sinopharm Chemical Reagent Co., Ltd. All chemicals were analytical-grade reagents and used as purchased without further purification. Rare earth chloride stock solutions were prepared by dissolving the corresponding metal oxide in hydrochloric acid at an elevated temperature.

### 2.2. Preparation of monodispersed polystyrene microspheres

Monodisperse PS colloidal microspheres were prepared by dispersion polymerization [35]. In a typical synthesis, the poly(*N*-vinylpyrrolidone) stabilizer (1.0 g) was dissolved in ethanol (38.2 ml) in a three-necked round bottom flask fitted with a condenser and a magnetic stirrer. The reaction vessel was then heated to 70°C under a nitrogen blanket and purged with nitrogen for 2 h. Then, a solution of azoisobutyronitrile (0.15 g) pre-dissolved in styrene monomer (15 g) was added to the reaction vessel with vigorous stirring. The styrene polymerization was allowed to proceed for 12 h before cooling to room temperature. The product was purified by repeated centrifugation and washed with ethanol. A white fine powder (PS) was finally obtained after being dried in a vacuum oven at 50°C.

### 2.3. Preparation of monodisperse PS@Lu(OH)CO_3_ microspheres

In the preparation procedure, 1 mmol of LuCl_3_ aqueous solution and the as-prepared PS microspheres (100 mg) were added to 50 ml deionized water and well dispersed with the assistance of ultrasonication for 30 min. Then, 2.0 g of urea was dissolved in the solution under vigorous stirring. Finally, the mixture was transferred into a 100 ml flask and heated at 90°C for 2 h with vigorous stirring before the product was collected by centrifugation. The precursors were washed by deionized water and ethanol three times.

### 2.4. Preparation of monodisperse hollow LuPO_4_ microspheres

In a typical synthesis, the as-obtained PS@Lu(OH)CO_3_ sample was dispersed in deionized water by ultrasonication for 30 min. Then, 0.2 g of NH_4_H_2_PO_4_ dissolved in 10 ml deionized water was dripped into the dispersion followed by further stirring. After additional agitation for 60 min, the as-obtained mixing solution was transferred into a Teflon bottle held in a stainless steel autoclave, sealed and maintained at 180°C for 12 h. As the autoclave was cooled to room temperature naturally, the precipitates were separated by centrifugation, washed with deionized water and ethanol in sequence, and then dried in air at 80°C for 12 h. The final hollow LuPO_4_ microspheres were obtained through a heat treatment at 800°C in air for 4 h with a heating rate of 1°C min^−1^. Hollow LuPO_4_:Ln^3+^ (Ln^3+ ^= Eu^3+^, Tb^3+^) spheres were prepared in a similar procedure except by adding corresponding Eu_2_O_3_, and Tb_4_O_7_ together with Lu_2_O_3_ as the starting materials as described above.

### 2.5. Characterization

The X-ray diffraction (XRD) patterns of the samples were recorded on a D8 Focus diffractometer (Bruker) with Cu-K*α1* radiation (*λ* = 0.15405 nm). Fourier transform infrared spectroscopy (FT-IR) spectra were measured with a Perkin–Elmer 580B infrared spectrophotometer with the KBr pellet technique. Thermogravimetric data were recorded with Thermal Analysis Instrument (SDT 2960, TA Instruments, New Castle, DE) with the heating rate of 10°C min^−1^ in an air flow of 100 ml min^−1^. The morphologies and composition of the as-prepared samples were inspected on a field emission scanning electron microscope (FESEM, SU8010, Hitachi). Low- to high-resolution transmission electron microscopy (TEM) was performed using FEI Tecnai G^2^ S-Twin with a field emission gun operating at 200 kV. Images were acquired digitally on a Gatan multiple CCD camera. The PL excitation and emission spectra were recorded with a Hitachi F-7000 spectrophotometer equipped with a 150 W xenon lamp as the excitation source. All measurements were performed at room temperature.

## 3. Results and discussion

**Scheme 1.**
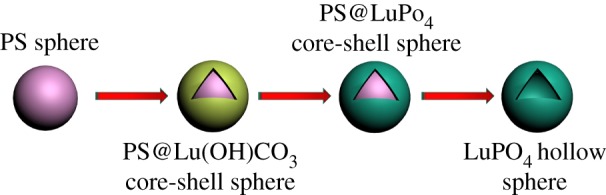
Schematic illustration of the possible growth mechanism of the LuPO_4_ hollow microspheres.

The synthesis protocol of the hollow LuPO_4_ microspheres is shown in scheme 1. In accordance with the previous reported method, the well-monodispersed PS colloidal microspheres were prepared by dispersion polymerization, and coated with a Lu(OH)CO_3_ layer using urea-based chemical precipitation to form a monodisperse core-shell PS@Lu(OH)CO_3_ microspheres (equations (3.1) and (3.2)). Subsequently, the core-shell PS@Lu(OH)CO_3_ microspheres were reacted with NH_4_H_2_PO_4_ under hydrothermal process, involving the replacement of the anions between CO_3_^2−^ and PO_4_^3−^ based on the Kirkendall effect [2,14]. The LuPO_4_ bumpy skin layer was constructed and assembled on the surface of the PS microspheres as the reaction progress (equation (3.3)). Finally, the PS microspheres were removed through calcination of the PS@LuPO_4_ sample and LuPO_4_ hollow microspheres were formed.

3.1 $$\begin{array}{r} \text{CO}{\left(\text{N}{\text{H}}_{2}\right)}_{2}+{\text{H}}_{2}\text{O}⇌\text{C}{\text{O}}_{2}+2\text{N}{\text{H}}_{3}, \end{array}$$

3.2 $$\begin{array}{r} \text{PS}+\text{L}{\text{u}}^{3+}+3\text{N}{\text{H}}_{3}+\text{C}{\text{O}}_{2}+2{\text{H}}_{2}\text{O}⇌\text{PS@Lu}(\text{OH})\text{C}{\text{O}}_{3}+3{\text{NH}}_{4}^{+} \end{array}$$

3.3 $$\begin{array}{r} \;\text{and}\;\text{PS@Lu}(\text{OH})\text{C}{\text{O}}_{3}+{\text{H}}_{2}{\text{PO}}_{4}^{-}⇌\text{PS@LuP}{\text{O}}_{4}. \end{array}$$

**Figure 1.**
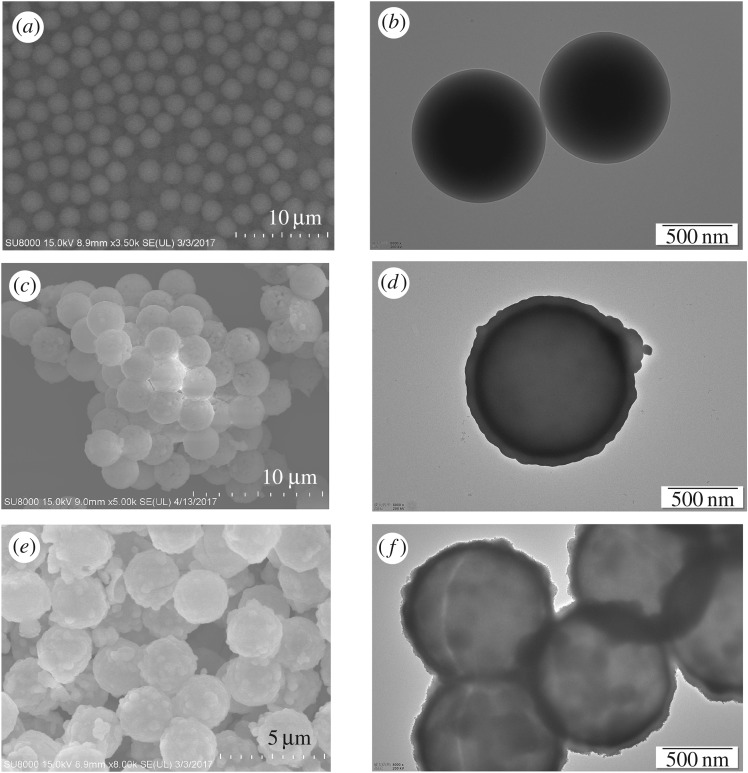
SEM and TEM images of (*a,b*) the PS spheres, (*c,d*) the core-shell PS@Lu(OH)CO_3_ microspheres and (*e,f*) the core-shell PS@LuPO_4_ microspheres.

Figure 1 shows SEM (left) and TEM (right) images of the PS microspheres (*a* and *b*), the core-shell PS@Lu(OH)CO_3_ microspheres (*c* and *d*), and the core-shell PS@LuPO_4_ microspheres (*e* and *f*). The as-obtained PS microspheres template consists of uniform microspheres with diameters of about 2.30 µm (figure 1*a*). It can be observed in the TEM image that the surfaces of monodisperse PS microspheres are very smooth (figure 1*b*). After deposition of Lu(OH)CO_3_ layer, the surface of PS@Lu(OH)CO_3_ core-shell microsphere is bumpier than PS microsphere. The size of the PS@Lu(OH)CO_3_ core-shell-structured microsphere (*ca* 2.50 µm) is larger than that of the bare PS microspheres (*ca* 2.4 µm) due to the amorphous Lu(OH)CO_3_ shell (figure 1*c*). From the TEM image (figure 1*d*), the core-shell structure can be easily found via the dark cores and the grey shell. Then, the core-shell-structured PS@LuPO_4_ microspheres with spiky LuPO_4_ shell were synthesized through an ion-exchange process under hydrothermal reaction conditions. The SEM image (figure 1*e*) shows that the core-shell PS@LuPO_4_ sample has an average size of about 2.55 µm and very rough surfaces with spikes. The TEM image (figure 1*f*) reveals that the sample with a diameter of 2.55 µm has a spiky surface and a solid structure, which agrees well with the SEM image.

**Figure 2.**
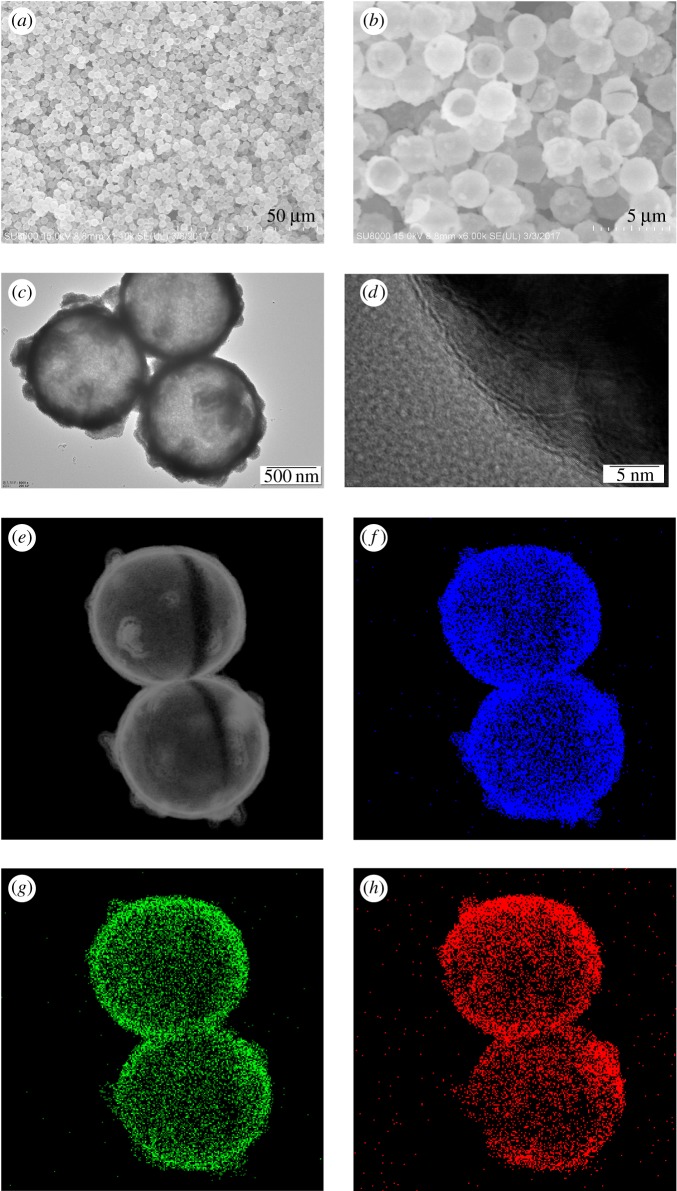
SEM (*a,b*), TEM (*c*), and HRTEM (*d*) images of the LuPO_4_ hollow microspheres. HAADF-STEM images of (*e*) the LuPO_4_ hollow microspheres, and the corresponding elemental maps for (*f*) Lu, (*g*) P and (*h*) O.

Figure 2 shows the SEM, TEM, HRTEM and HAADF-STEM images of the LuPO_4_ sample. The low- and high-magnification SEM images in figure 2*a,b* show that monodisperse and uniform LuPO_4_ hollow microspheres can be prepared by this approach. The size of the hollow microspheres is about 2.45 µm, which implies that the PS templates essentially determine the shape and structure of the final products. A small quantity of broken hollow microspheres further imply that the LuPO_4_ microspheres have hollow structures because of the release of gaseous carbon/nitrogen oxides and water when the oxidation process of PS microspheres occurred during the calcination process. We also can see that the wall thickness of the hollow microspheres is about 75 nm. To provide further insight into the LuPO_4_ hollow microspheres, a TEM investigation was performed. The TEM image (figure 2*c*) of the LuPO_4_ hollow microspheres exhibits spherical morphology and a spiky surface. The strong contrast between the dark edge and the pale centre is direct evidence of the hollow nature of the microspheres. The average size of the hollow spheres and the thickness of the shells are estimated to be about 2.45 µm and 75 nm, respectively, which is in good agreement with the SEM observations. The obvious lattice fringes in the HRTEM image (figure 2*d*) confirm the high crystallinity. The interplanar distance between the adjacent lattice fringes is 0.253 nm, which can be indexed as the *d* spacing of the (112) plane of LuPO_4_ crystal. Elemental mapping of the LuPO_4_ hollow microspheres indicates that Lu, P and O elements are evenly distributed on the LuPO_4_ hollow microspheres (figure 2*e–h*).

**Figure 3.**
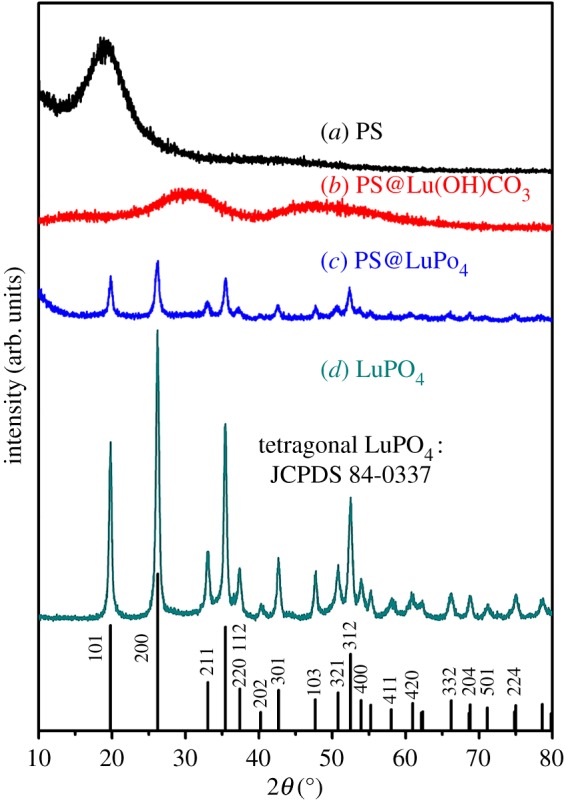
XRD patterns of (*a*) the PS spheres, (*b*) the core-shell PS@Lu(OH)CO_3_ microspheres, (*c*) the core-shell PS@LuPO_4_ microspheres and (*d*) the LuPO_4_ hollow microspheres.

Figure 3 shows the XRD results of the PS spheres, the core-shell PS@Lu(OH)CO_3_ microspheres, the core-shell PS@LuPO_4_ microspheres and the final LuPO_4_ hollow microspheres. In figure 3*a* for the PS spheres, an obviously broadened diffraction peak at 19° is observed, which can be assigned to the typical XRD pattern of PS spheres [10,36]. For the core-shell PS@Lu(OH)CO_3_ microspheres (figure 3*b*), two broad bands at 30° and 47° can be observed, which imply that the as-formed core-shell PS@Lu(OH)CO_3_ microspheres are amorphous. Figure 3*c* shows the XRD pattern of the sample that the core-shell PS@Lu(OH)CO_3_ microspheres were treated with NH_4_H_2_PO_4_ in the hydrothermal process. All of the diffraction peaks can be indexed as pure tetragonal phase, and coincide well with the standard data of LuPO_4_ (JCPDS No. 84-0337). It means the product is the core-shell PS@LuPO_4_. After annealing of the core-shell PS@LuPO_4_ microspheres at 800°C for 4 h, the position of all peaks does not change (figure 3*d*). However, the diffraction peaks become stronger and sharper due to the increase of crystallinity. This is important for phosphors, because high crystallinity generally means less traps and stronger luminescence. Thus, it can be concluded that the calcination process has a dual function: elimination of the PS spheres cores to form hollow microspheres and increase of crystallinity.

**Figure 4.**
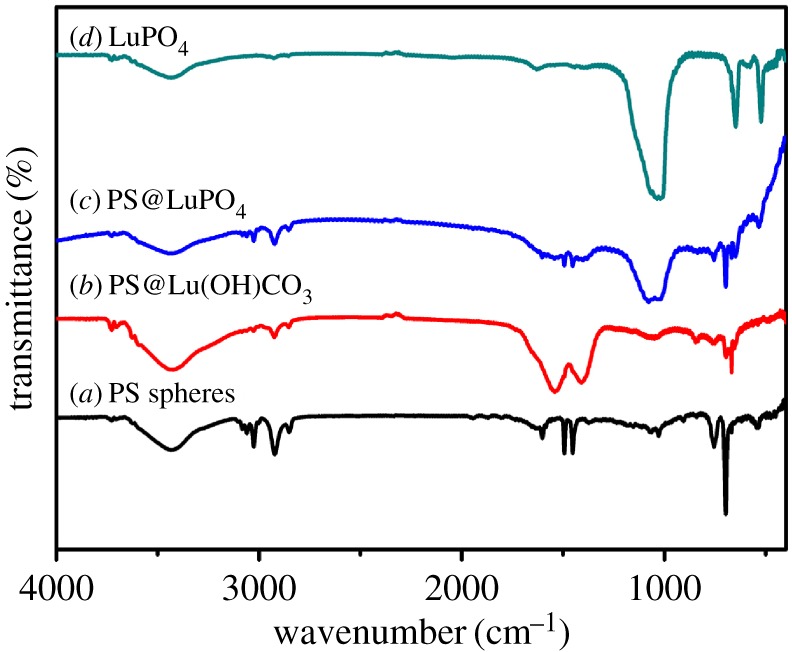
FT-IR spectra of (*a*) the PS spheres, (*b*) the core-shell PS@Lu(OH)CO_3_ microspheres, (*c*) the core-shell PS@LuPO_4_ microspheres and (*d*) the LuPO_4_ hollow microspheres.

**Figure 5.**
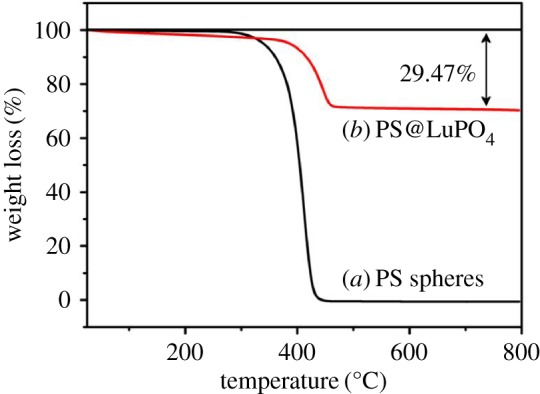
TGA curves of (*a*) the PS spheres and (*b*) the core-shell PS@LuPO_4_ microspheres.

To further examine the chemical compositions of the samples, FT-IR spectroscopy was conducted for all the products (figure 4). For the PS microspheres (figure 4*a*), the characteristic adsorption peaks at about 3150–2800, 1650–1350 and 600–820 cm^−1^ can be attributed to the stretching vibrations of aromatic C–H in-plane, stretching vibrations of aromatic C–C and bending vibrations of aromatic C–C out-of-plane, respectively [37]. Compared with the PS microspheres, the core-shell PS@Lu(OH)CO_3_ microspheres not only exhibit characteristic absorption bands of the PS microspheres, but also show the bands at 1543, 1408, 838, 760, 698 and 664 cm^−1^, corresponding to CO(*v*_as_), CO (*v*_s_), CO (*δ*), OH-- (*δ*) and CO (*δ*) (*v*_as_ = asymmetric stretch; *v*_s_ = symmetric stretch; *δ *= deformation) (figure 4*b*) [10,36]. This result further indicates that the compositions of the core-shell PS@Lu(OH)CO_3_ are the PS microspheres and Lu(OH)CO_3_. For the core-shell PS@LuPO_4_ microspheres (figure 4*c*), the vibration bands at 648 and 1023 cm^−1^ represent the characteristic adsorption of the phosphate groups [14,38]. It means that the core-shell PS@Lu(OH)CO_3_ microspheres can convert to the core-shell PS@LuPO_4_ microspheres during the hydrothermal process. In the FT-IR spectrum of the LuPO_4_ hollow microspheres, all of the functional groups of the PS microspheres nearly disappear, and the characteristic adsorptions of the phosphate groups do not change, which demonstrates that the PS template can thoroughly be removed by calcination. We also studied the thermal behaviour of the PS spheres and the core-shell PS@LuPO_4_ microspheres by thermogravimetric analysis (TGA) technique (figure 5). There is one stage of weight loss for the PS spheres (line *a*), which can be attributed to the splitting burning of the PS spheres. For the core-shell PS@LuPO_4_ microspheres, there are two stages of weight loss (line *b*): One is a slow weight loss because of the dehydration and densification of the PS microspheres. The other one is the burning of the PS microspheres. Finally, the residual weight percentage is about 70.53%, which accounts for the final LuPO_4_ hollow microspheres, suggesting the considerably high yield of the hollow phosphors prepared by this method.

**Figure 6.**
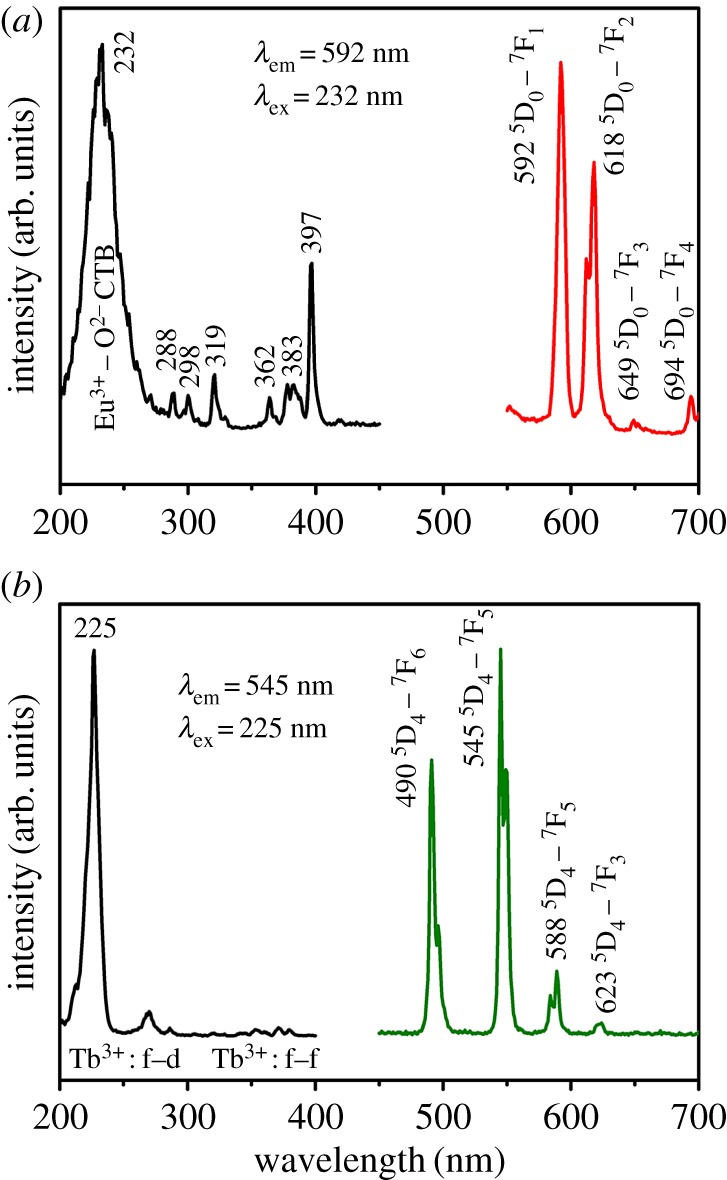
Excitation (left) and emission (right) spectra of (*a*) LuPO_4_: 5 mol% Eu^3+^ and (*b*) LuPO_4_: 5 mol% Tb^3+^ hollow microspheres.

Figure 6*a* shows the excitation and emission spectra of the LuPO_4_:Eu^3+^ hollow microspheres. The excitation spectrum is composed of a broadband from 200 to 280 nm and some sharp peaks from 280 to 450 nm. The broad excitation band can be ascribed to the charge transfer band between the O^2−^ anions and the Eu^3+^ ions. The other peaks are attributed to the f → f transitions within the Eu^3+^ 4f^6^ electron configuration. Upon excitation at 232 nm, the emission spectrum consists of the ^5^D_0_ → ^7^F*_J_* (*J* = 1, 2, 3, 4) transition lines of the Eu^3+^ ions. The strongest orange-red emission arises from the forced magnetic-dipole ^5^D_0_ → ^7^F_1_ (592 nm) transition of the Eu^3+^ ions. All the other emission peaks can be assigned to the ^5^D_0_ → ^7^F_2_ (618 nm), ^5^D_0_ → ^7^F_3_ (649 nm) and ^5^D_0_ → ^7^F_4_ (694 nm) transitions of Eu^3+^ ions, respectively.

Figure 6*b* depicts the excitation and emission spectra of the LuPO_4_:Tb^3+^ hollow microspheres. The excitation spectrum is composed of two bands with a maximum at 225 and 270 nm due to the f → d transitions of Tb^3+^ in the LuPO_4_ lattice, and some weak lines in the longer wavelength. The peaks with weaker intensity are assigned to the transitions from the ^7^F_6_ ground state to the different excited states of the Tb^3+^ ions, that is, ^5^G_2_ (350 nm), ^5^D_2_ (354 nm), ^5^G_6_ (370 nm) and ^5^D_3_ (381 nm), respectively. Upon the excitation at 225 nm, the obtained emission spectrum exhibits four obvious lines centred at 490, 545, 588 and 623 nm, that can be attributed to the transitions from the ^5^D_4_ excited state to the ^7^F*_J_*(*J* = 6, 5, 4, 3) ground states of the Tb^3+^ ions, respectively. The ^5^D_4_ → ^7^F_5_ transition at 545 nm is the most prominent group.

## 4. Conclusion

In summary, well-dispersed and homogeneous LuPO_4_ hollow microspheres have been successfully achieved by the combination of a facile homogeneous precipitation approach, an ion-exchange process and a calcination process. The possible mechanism for the overall formation process of the hollow microspheres has been discussed in detail. Under UV excitation, the LuPO_4_:Ln^3+^ (Ln^3+^ = Eu, Tb) hollow microspheres exhibit orange-red and green emissions, respectively. Furthermore, it is expected that our synthetic approach may open a new way for monodisperse hollow microspheres that exhibit promising physicochemical properties.

## Data accessibility

This paper does not have any additional data.
